# Symptom burden, fatigue, sleep quality and perceived social support in hemodialysis patients with musculoskeletal discomfort: a single center experience from Egypt

**DOI:** 10.1186/s12891-023-06910-z

**Published:** 2023-10-04

**Authors:** Mohammed Kamal Nassar, Samar Tharwat, Sara M. Abdel-Gawad, Rabab Elrefaey, Alaa A. Elsawi, Abdelrahman Mohamed Elsayed, Eman Nagy, Shimaa Shabaka, Rasha Samir Shemies

**Affiliations:** 1 Mansoura Nephrology & Dialysis Unit (MNDU), Faculty of Medicine, Mansoura University, El Gomhurria St, Mansoura, 35516 Egypt; 2 Rheumatology & Immunology Unit, Department of Internal Medicine, Faculty of Medicine, Mansoura University, Mansoura, Egypt; 3 Faculty of Medicine, Mansoura University, Mansoura, Egypt

**Keywords:** Musculoskeletal disorders (MSDs), End stage kidney disease, Hemodialysis, Fatigue, Symptom burden, Sleep quality, Social support, Nordic Musculoskeletal Questionnaire (NMQ-E)

## Abstract

**Background and aims:**

Musculoskeletal disorders (MSDs) are commonly encountered in hemodialysis (HD) patients. However, the causes linked to these disorders are still partially defined. The aim of this study was to determine the frequency of MSDs and their relationship to a variety of clinico-social characteristics such as sleep quality, mood disorders, fatigue, and social support, in addition to the patients’ clinical and therapeutic profile.

**Method:**

The study included 94 patients on maintenance HD. Clinical and Sociodemographic data was gathered. To investigate the prevalence and trends of MSDs, the Nordic Musculoskeletal Questionnaire (NMQ-E) was employed. Patients completed the modified Edmonton Symptom Assessment System, Pittsburgh Sleep Quality Index (PSQI), multidimensional Fatigue Inventory (MFI-20), and Perceived Social Support from Family Scales. Univariate and multivariate regression analysis were used to assess the determinants of MSDs.

**Results:**

The patients' mean age was 49.73 and 59.6% were males. Seventy-two percent of patients were afflicted by MSDs. Knee pain (48.9%), low back pain (43.6%), shoulder pain (41.6%), hip/thigh pain (35.1%), and neck pains (35.1%) were the most reported MSD domains. Pain (*p* = 0.001), fatigue (*p* = 0.01), depression (*p* = 0.015), and anxiety (*p* = 0.003) scores were substantially higher in patients with MSDs. Furthermore, patients with MSDs engaged in less physical activity (*p* = 0.02) and perceived less social support (*p* = 0.029). Patients with MSDs had lower subjective sleep quality, daytime dysfunction domains, and global PSQI scores (*p* = 0.02, 0.031, 0.036, respectively). Female gender (*p* = 0.013), fatigue (*p* = 0.012), depression (*p* = 0.014), anxiety (*p* = 0.004), lower activity (*p* = 0.029), and PSQI score (0.027), use of erythropoiesis-stimulating agents (ESAs), antihypertensive drugs, calcium and Iron supplementation were all significantly associated with MSDs. At the multivariable regression model, administration of ESAs (*p* = 0.017) and pain score (*p* = 0.040) were the only independent variables associated with the outcome.

**Conclusion:**

MSDs are quite common among HD patients. Female gender, pain, fatigue, depression, anxiety, reduced activity, poor sleep quality, and use of ESAs are all significantly associated with MSDs in HD patients. Patients with MSD perceived less social support compared to the other group. Patients treated with antihypertensive drugs, calcium and iron supplements were more likely to suffer MSDs.

## Introduction

Musculoskeletal disorders (MSDs) impair the quality of life in patients with end stage kidney disease (ESKD) [1, 2], potentially leading to major disabilities and functional losses [3, 4]. Dialysis related MSDs have been reported in more than 70% of patients on dialysis [5, 6]. Moderate to severe MSK pain affects approximately half patients on HD [7, 8]. This applies to patients on either hemodialysis or peritoneal dialysis due to a variety of causes that can be related to the primary kidney disease itself or the complications of the modality of dialysis [7]. The majority of HD patients were found to have more than one site and kind of musculoskeletal (MSK) manifestations [9]. Many patients suffer multiple concurrent symptoms or symptom clusters. Symptom clusters refer to relevant concurrent symptoms that almost share a common etiology. Upper and lower extremity MSK complications as well as osteoporosis were frequently observed in HD patients [2, 10]. Patients with ESKD may present with pain related to renal bone disease (osteitis fibrosa cystica, osteomalacia), and calciphylaxis [11]. Dialysis related arthropathy is another clinical, biological, and radiological entity including articular, vertebral and bone problems as well as carpal tunnel syndrome [6]. Several pathogenetic mechanisms contribute to the occurrence of MSK complications in HD patients, including hyperparathyroidism, renal osteodystrophy, β2-microglobulin amyloidosis, and uremic myelopathy [1]. The pathophysiological interactions of chronic kidney disease-mineral and bone disorders (CKD-MBD) including vit D deficiency, phosphate retention and hyperparathyroidism greatly predispose to MSK disease in patients with ESKD. Physical inactivity in dialysis patients is another risk factor for MSDs particularly osteoarthritis and accelerated deterioration of motor functions [6, 12]. Protein energy wasting (PEW), is an additional key prognostic indicator particularly with the increasing pool of the elderly undergoing HD [13]. The psychosocial correlates of MSDs in HD patients have not been adequately studied. On a general basis, MSDs have been strongly linked to depression and anxiety. Similarly, psychological distress and depression worsen musculoskeletal pain displaying a vicious cycle of worsening pain and low mood [14]. Sleep quality is thought to predict pain rather than pain predicts sleep quality [15]. It has been revealed that sleep deprivation alters pain processing, inducing hyperalgesia and contributing to acute and chronic pains [15, 16]. MSK pain is also found to be associated with loneliness and perceived insufficiency of social support [17, 18]. We hypothesize that these clinical and psychosocial factors contribute as well to MSDs in patients on HD. The present study aimed to study the prevalence of MSK symptoms among patients on HD and identify their association with many clinicosocial characteristics including quality of sleep, social support, and fatigue.

## Methods

### Study design and population

**Fig. 1:**
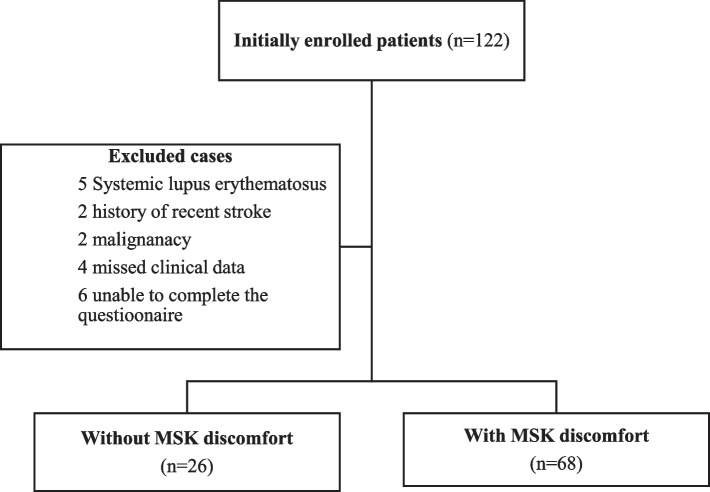
The study flowchart

This study is a cross-sectional study which was conducted at Mansoura nephrology and dialysis unit, Mansoura University, Egypt in 2022. One hundred and twenty-two patients satisfied all the requirements to be included. Of them, 18 were excluded; five patients with a diagnosis of systemic lupus erythematosus, two with a recent history of stroke, two had active malignancy, four with missed clinical data and six were unable to complete the questionnaire. Finally, 94 patients were included in the study. The flowchart illustrating the participants selection is demonstrated in Fig. 1. The sample included 94 ESKD patients on regular HD for at least 3 months duration who gave informed consent to be enrolled in the study. Patients who had a history of chronic rheumatic or muscular diseases such as systemic lupus erythematosus, rheumatoid arthritis, seronegative arthropathy or fibromyalgia prior to initiating HD treatment were not eligible to participate. In addition, those who had a concomitant illness that influenced the musculoskeletal and neurological system, such as active malignancy or chronic neurological disorder, were excluded from the study. Participants with incomplete data were also ruled out from the study. All participants received standard dialysis prescription according to the recommendations of the Egyptian ministry of health with a regimen of thrice weekly sessions, 4 h each. Bicarbonate based dialysis solutions with a dialysate flow rate of 500 ml/min and a dialyzer surface area of 1.3–2.2 m^2^ were utilized.

### Ethical consideration

The study was approved by Mansoura Faculty of Medicine Institutional Research Board “MFM-IRB", (Approval No: R.22.11.1944) and it adhered to the guidelines outlined in the declaration of Helsinki in 1995. All participants were given detailed information about the study. Informed consents were obtained from the participants themselves or from the legal guardians of uneducated patients. The present study applied the Strengthening the Reporting of Observational Studies in Epidemiology) statement (STROBE) guidelines during the manuscript preparation process.

### Sample size and sampling procedure

Based on previous Egyptian studies by Afifi et al. [19] and Ezzat et al. [2], we hypothesized an 83% prevalence of musculoskeletal manifestations (MSM) among HD patients. Sample size (*n*) was calculated by the following formula [20]:

$$n=\frac{{z}^{2}\times P\times \left(1-P\right)}{{d}^{2}}$$

A total sample size of 93 HD patients achieves an 80% confidence level (z = 1.282) for an expected prevalence (P) of 0.83 and an acceptable margin of error (d) of ± 0.05.

This sample size is sufficient to run univariable binary logistic regression to predict MSM in HD patients [21].

### Survey and questionnaire administration

The questionnaire was developed based on extensive review of the literature [22–27]. Following editing and review, six medical staff members reviewed the questionnaires’ design, substance, language, and ease of completion. Based on this, four new items were added, two were eliminated, and two were re-worded.

### Questionnaire administration

The study was conducted at Mansoura Nephrology and dialysis unit through interview questionnaires. This style of questioning enables more complex issues to be explored than the self-administered style and provides more detailed explanations of the queries. Each interviewer conducted a structured face-to-face interview with each patient, presenting the items orally, using easy, understandable language. It was estimated that it would take between 15 and 20 min to complete. The interviewer attempted to speak with as many HD patients as possible. All HD patients who opted to participate were questioned during one of their regular hemodialysis sessions. S.A, R.E, A.A.E, E.N, A.M.E and S.S. conducted the interviews. Participants' identity and confidentiality were protected by not requesting personal information.

### Variables gathered

#### Sociodemographic and clinical data

Data was obtained from the medical records of all patients. The sociodemographic characteristics (Special habits, occupation, level of education, socioeconomic status, and lifestyle), clinical, and therapeutic data of the included patients were recorded. The included patients filled in the Nordic Musculoskeletal Questionnaire (NMQ-E), the modified Edmonton Symptom Assessment System, multidimensional Fatigue Inventory (MFI-20), Pittsburgh Sleep Quality Index (PSQI), and multi-dimensional scale of Perceived Social Support. All the questionnaires used have been formerly validated [22–27]. The Nordic Musculoskeletal Questionnaire (NMQ-E) has been translated into Arabic.

#### The Nordic Musculoskeletal Questionnaire (NMQ-E)

The Nordic Musculoskeletal Questionnaire (NMQ-E) was basically used to assess the presence and distribution of MSK manifestations thoroughly at different body parts. The NMQ-E questionnaire includes a picture, in which the patient can see and define the approximate position of the body parts, he has or has had a trouble with [22]. The investigated symptom sites include neck, shoulders, upper back, elbows, low back, wrist/hands, hips/thighs, knees, and ankles/feet. Respondents are asked if they have had any musculoskeletal trouble in the last 7 days and last 12 months which has prevented normal activity. Additional questions relating to the neck, the shoulders and the lower back consider any accidents affecting each area, functional impact at home and work (change of job or duties), duration of the problem, assessment by a health professional and musculoskeletal problems in the last 7 days [23]. The NMQ-E questionnaire has been translated into Arabic to be clearly understood by the patients. The pre-final version of the translated questionnaire was pilot tested on a small sample before final approval. Patients were verbally asked by an interviewer to confirm their responses.

#### The modified Edmonton Symptom Assessment System (Modified ESAS)

The modified Edmonton Symptom Assessment System, a questionnaire which has been validated for simple and rapid documentation of symptom burden in patients with advanced disease [24, 28], was used to assess symptom severity for pain, tiredness, lack of appetite, nausea, shortness of breath, drowsiness, depression, anxiety, sleep, and general well-being. The patients gave a score according to the severity of their symptoms using a 10-point Likert scale ranging from 0 (no symptom) to 10 (worst possible symptom). Patients who gave scores of 7–10 were considered to have severe symptoms, while scores of 4–6 were representative of moderate symptoms.

#### The multidimensional Fatigue Inventory (MFI-20)

The multidimensional Fatigue Inventory (MFI-20) is a 20-item scale created for evaluation of five aspects of fatigue including general, physical, and mental fatigue, in addition to reduced motivation, and activity [25]. The patients used a scale ranging from 1–7 to indicate the severity of their symptoms. Higher MFI scores indicated a high degree of fatigue.

#### Pittsburgh Sleep Quality Index (PSQI)

Pittsburgh Sleep Quality Index (PSQI) is a tool which was designed to assess the usual sleep habits for one previous month. It includes 19 self-rated questions, combined to form seven-component-scores. These components include subjective sleep quality, sleep latency, sleep duration, habitual sleep efficiency, sleep disturbances, use of sleep medications, and daytime dysfunction [26]. The patients gave a score of 0–3 for each component based on the difficulty of sleep. A total score of 0–21 reflects the difficulty of sleep in the entire components, higher scores correspond to poorer sleep quality and more difficulty of sleep. Extra five questions were rated by the patients’ bed partners if available and were not included in the scoring.

#### The multi-dimensional scale of Perceived Social Support (MSPSS)

The included patients eventually responded to the multi-dimensional scale of Perceived Social Support, a 12-item questionnaire designed to identify an individual’s perceived level of social support with family, friends, and significant others. Each item uses a seven-point Likert scale ranging from 1 (very strongly disagree) to 7 (very strongly agree). The higher the score, the greater the social support the patient perceives [27].

#### Blood sampling and laboratory tests

Just prior to the beginning of the first HD session of the week, blood samples were taken from the arteriovenous fistulae (AVF). The standard laboratory tests including serum calcium, phosphorus, albumin, parathyroid hormone, ferritin, and transferrin saturation in addition to the full blood counts were carried out on the days of the blood sampling, using the automatic analyzer.

#### Statistical analysis

This study used statistical software of IBM SPSS version 29.0 to analyze the data. The patients’ demographic and clinical characteristics were analyzed using a descriptive statistical analysis. Categorical data were presented by frequency tables (Number and percentages). Medians (min–max) and means and standard deviation (SD) were used for all quantitative values. The distribution of continuous variables was examined with SK test for normality. The significance of differences between continuous variables was determined with independent samples t- test for normally distributed variables and Mann–Whitney test for not normally distributed variables, as appropriate. Chi square or Fisher’s exact test were used to test the significance of categorical data. Univariable logistic regression was performed to study the association between the clinical and laboratory data included and the MSK complications in HD patients. Odds Ratio (OR) of its 95% Confidence Interval (CI) was calculated. The results were considered significant when the probability of error is less than 5% (p ≤ 0.05). A multivariable regression model with forward selection procedure was performed including variables with *p*-value < 0.05 in the univariate analysis.

## Results

### Sociodemographic and clinical characteristics

Out of 122 patients with ESKD on regular hemodialysis for a minimum duration of three months who fulfilled the inclusion requirements, 94 patients were included. The mean age of the studied patients was 49.73 ± 15.88 years and more than half of the included sample (59.6%) were males. MSDs affected nearly three quarters (72.3%) of the included patients. Hypertension was more frequently encountered in patients with MSDs (64.7%). MSDs were more significantly reported in patients regularly receiving Erythropoiesis stimulating agents (ESAs), calcium, iron supplements, and antihypertensive drugs (*p* < 0.005, 0.006, 0.007, 0.001 respectively). No significant statistical difference was found as regards the studied laboratory parameters between patients with and without MSDs**.** Other sociodemographic and clinical characteristics of the studied patients are shown in Table 1.

**Table 1:** Sociodemographic and clinical characteristics and therapeutic data of participants (*n* = 94)

**Variable**	**Total** (*n* = 94)	**Without MSK discomfort** ***n* = 26** **(27.7%)**	**With MSK discomfort** ***n* = 68** **(72.3%)**	**Measure of Effect**	***P***
*Sociodemographic characteristics*					
*Gender*^a^*, n (%)*				-0.267	
Male	56 (59.6)	21 (80.8	35 (51.5)		0.010*
Female	38 (40.4)	5 (19.2)	33 (48.5)		
Age, mean ± SD,years	49.73 ± 15.88	46.33 ± 18.95	50.49 ± 15.17	-0.262	*0.437*
*Smoking*^a^*, n (%)*				0.114	
Never	*80* (85.1)	*21* (80.8)	*59* (86.8)		0.543
Former smoker	*13* (13.8)	*5* (19.2)	*8* (11.8)		
Current smoker	*1* (1.1)	*0*	*1* (1.5)		
*Level of education*^a^*, n (%)*				0.310	
Not educated	9 (9.6)	0	9 (13.2)		0.108
Low School	7 (7.4)	1 (3.8)	6 (8.8)		
Middle School	27 (28.7)	12 (46.2)	15 (22.1)		
High School	37 (39.4)	9 (34.6)	28 (41.2)		
College degree	12 (12.8)	4 (15.4)	8 (11.8)		
Post-graduate	2 (2.1)	0	2 (2.9)		
*Occupation*^a^*, n (%)*				0.282	
Not employed	51 (54.3)	18 (69.2)	33 (48.5)		0.058
Employed	19 (20.2)	6 (23.1)	13 (19.1)		
Retired	9 (9.6)	2 (7.7)	7 (10.3)		
Not able to work due to disability	15 (16)	0	15 (22.1)		
Active lifestyle^a^*, n (%)*	40 (42.6)	14 (53.8)	26 (38.2)	-0.141	0.171
*Socioeconomic status*^a^*, n (%)*				0.234	
Low	48 (51.1)	18 (69.2)	30 (44.1)		0.076
Average	43 (45.7)	7 (26.9)	36 (52.9)		
High	3 (3.2)	1 (3.8)	2 (2.9)		
***Anthropometric measures***					
Weight, mean ± SD,Kg	78.93 ± 19.03	72.64 ± 20.27	80.27 ± 18.64	-0.403	0.212
Height, mean ± SD,m	1.67 ± 0.09	1.69 ± 0.09	1.65 ± 0.09	0.246	0.398
BMI, mean ± SD,Kg/m^2^	28.24 ± 6.54	25.26 ± 5.61	29.83 ± 6.58	-0.562	0.081
***Clinical characteristics***					
Duration of hemodialysis,median (min–max),years	3.1 (0.25–22)	1.5 (0.5–13)	4 (0.25–22)	0.204	0.067
*Associated comorbidities, n (%)*					
Diabetes mellitus^b^	10 (10.6)	1 (3.8)	9 (13.2)	0.136	0.275
Hypertension^a^	49 (52.1)	5 (19.2)	44 (64.7)	0.407	< 0.001*
Ischemic heart disease^a^	7 (7.4)	1 (3.8)	6 (8.8)	0.085	0.669
***Therapeutic data****, n (%)*					
Erythropoitin	59 (62.8)	7 (26.9)	52 (76.5)	**0.458**	< 0.001*
Calcium supplementation^a^	47 (50)	7 (26.9)	40 (58.8)	**0.285**	< 0.006*
Iron supplementation^a^	39 (41.5)	5 (19.2)	34 (50)	**0.279**	< 0.007*
Antihypertensives^b^	41 (43.6)	4 (15.4)	37 (54.4)	**0.352**	< 0.001*
Antidiabetics^b^	9 (9.6)	1 (3.8)	8 (11.8)	**0.120**	0.436
***Laboratory data***					
HB ^c^, mean ± SD, g/dL	10.94 ± 1.43	10.46 ± 1.33	11.08 ± 1.44	-0.441	0.081
Ferritin^d^, median (min–max), ng/mL	275.5 (12.4–1730)	316.3 (21.9–848.4)	270.2 (12.4–1730)	0.031	0.777
TSAT^d^, median (min–max),%	21 (5–59)	21 (5–56)	21 (7–59)	0.069	0.525
Serum albumin^c^ mean ± SD, g/dL	3.91 ± 0.35	3.88 ± 0.41	3.92 ± 0.34	0.061	0.567
Ca^c^ mean ± SD, mg/dL	8.37 ± .872	8.47 ± 0.57	8.34 ± 0.95	-0.112	0.435
PO4^d^ median (min–max), mg/dL	5.1 (1.9–10.7)	5 (10–10.7)	5.6 (2–10.2)	0.103	0.339
PTH^d^ median (min–max), pg/mL	401 (4.6–1978)	422 (36.9–1467)	397 (4.6–1978)	0.056	0.605
Urea reduction ratio (URR)	60.66 ± 11.49	59.84 ± 9.04	60.92 ± 12.21	-0.093	**0.711**
Kt/v URR	1.23 ± 0.45	1.19 ± 0.36	1.24 ± 0.48	-0.092	**0.713**
Kt/v Duagirdas	1.18 ± 0.41	1.13 ± 0.30	1.19 ± .44	-0.157	**0.533**

*BMI* Body Mass Index, *HB* hemoglobin, *TSAT* Transferrin saturation, *Ca* calcium, *PO4* phosphate, *PTH* parathormone

^*^P < 0.05,^a^Chi square test,^b^Fisher's exact test, ^c^independent sample t-test,^d^Mann–Whitney test

### Distribution and determinants of musculoskeletal complications

**Fig. 2:**
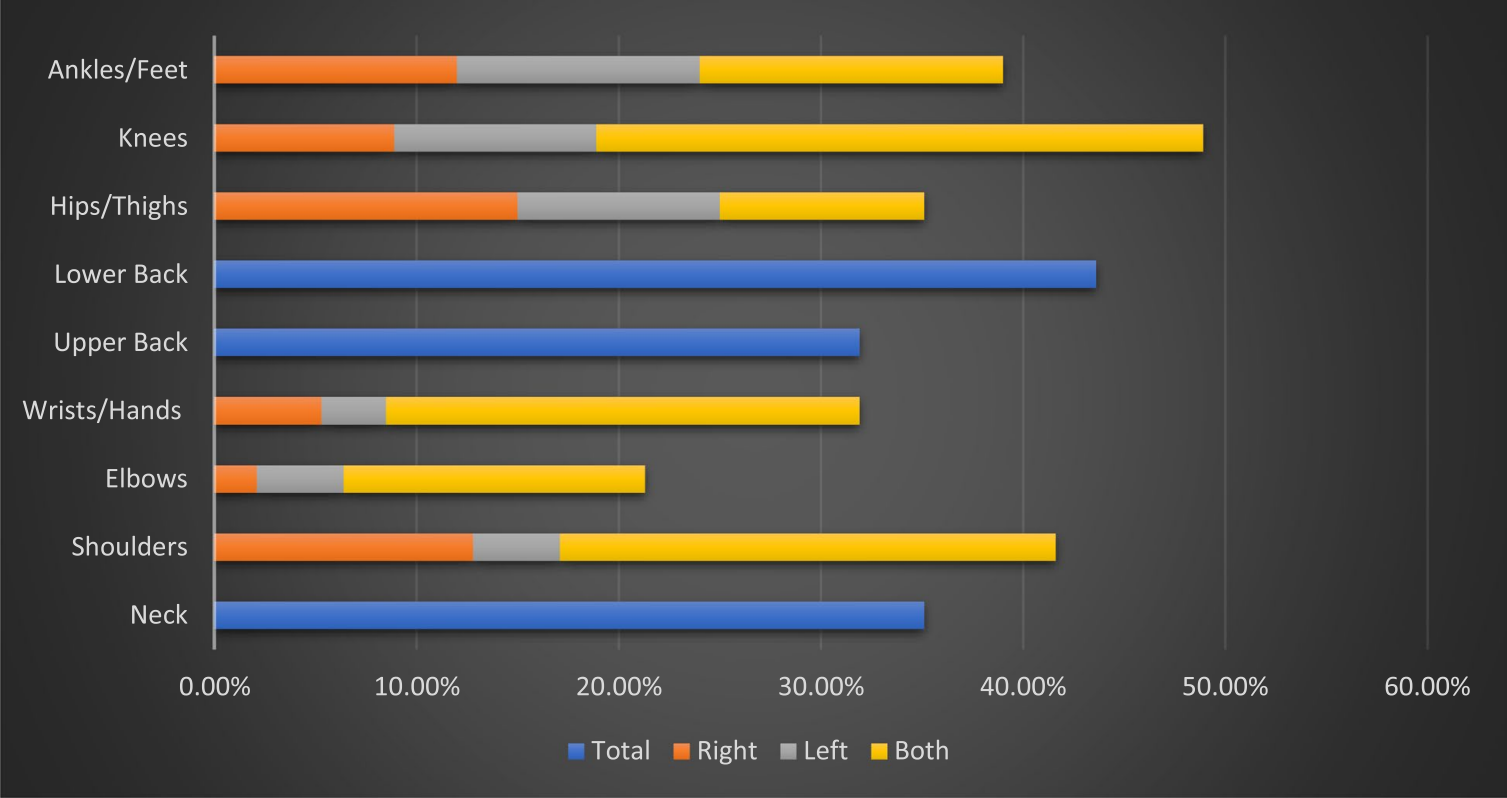
Distribution of MSK discomfort among the studied HD patients (*n* = 94)

The most frequently encountered MSD domains were knee pain (48.9%), low back pain (43.6%), shoulder pain (41.6%), hip/thigh pain (35.1%) and neck pain (35.1%) (Fig. 2). Regarding Pain and symptom burden, patients with MSK discomfort were more likely to present with pain and fatigue (*p* = 0.001 and 0.01 respectively). Depression, anxiety, and drowsiness were more frequently reported in patients with MSK discomfort compared to patients who did not report MSDs (*p* = 0.015, 0.003, and 0.014 respectively).

According to the multidimensional Fatigue Inventory (MFI-20), patients with MSDs experienced significantly reduced activity (*p* = 0.02) in comparison to the other group. Using Pittsburgh Sleep Quality Index (PSQI) to assess the usual sleep habits it has been revealed that patients with MSDs had worse scores as regard Subjective sleep quality, and daytime dysfunction (*p* = 0.02, and 0.031, respectively). Moreover, the global PSQI score was worse among patients with MSDs (*p* = 0.036). Regarding the multi-dimensional scale of Perceived Social Support, patients with MSDs had lower total MSPSS scores denoting less social support (*p* = 0.029), however, no significant statistical difference has been found between patients with and without MSDs as regards the subscales: family, friends, and significant other subscales. It is noteworthy mentioning that patients with MSDs had less social support from friends, however, this was non-statistically significant (Table 2).

**Table 2:** Symptom burden, fatigue, sleep quality and perceived social support in patients with and without MSK discomfort in HD patients

**Variable**^**a**^	**Total** (*n* = 94) median (min–max)	**Without MSK discomfort** ***n* = 26** median (min–max)	**With MSK discomfort** ***n* = 68** median (min–max)	Measure of effect	***P***
***Modified (ESAS)***					
Pain	4 (0–10)	0 (0–8)	4.5 (0–10)	**0.388**	< 0.001*
Fatigue	3 (0–9)	1 (0–6)	4 (0–9)	**0.273**	0.010*
Nausea	0 (0–10)	0 (0–7)	1 (0–10)	**0.168**	0.114
Depressed	3 (0–8)	0 (0–7)	4 (0–8)	**0.259**	0.015*
Anxiety	3 (0–10)	0 (0–7)	4 (0–10)	**0.315**	0.003*
Drowsiness	3 (0–10)	0 (0–7)	4 (0–8)	**0.259**	0.014*
Shortness of breath	2 (0–9)	0 (0–7)	3 (0–9)	**0.167**	0.114
Best appetite	4 (0–10)	3 (0–9)	5 (0–10)	**0.173**	0.103
Best sleep	5 (0–10)	4 (0–9)	5 (0–10)	**0.094**	0.377
Best feeling or wellbeing	4 (0–10)	4 (0–10)	4 (0–10)	**0.151**	0.153
**Multidimensional Fatigue Inventory**					
General fatigue	10 (6–16)	10 (7–13)	10 (6–16)	0.069	0.603
Physical fatigue	11 (4–20)	12 (6–15)	11 (4–20)	0.046	0.706
Reduced activity	10 (4–20)	9 (6–12)	11 (4–20)	0.298	0.020*
Reduced motivation	10 (4–18)	9 (5–12)	10 (4–18)	0.238	0.083
Mental fatigue	9 (3–16)	8 (5–13)	10 (3–16)	0.164	0.237
***Pittsburgh Sleep Quality Index***					
Subjective sleep quality	1 (0–3)	1 (0–3)	1 (0–3)	**0.249**	0.020*
Sleep latency	2 (0–3)	2 (0–3)	2 (0–3)	**0.083**	0.438
Sleep duration	2 (0–3)	2 (0–3)	2 (0–3)	**0.080**	0.436
Habitual sleep efficiency	1 (0–3)	1 (0–3)	1 (0–3)	**0.048**	0.640
Sleep disturbances	1 (0–3)	1 (0–2)	1 (0–3)	**0.177**	0.086
Use of sleep medications	0 (0–3)	0 (0–3)	0 (0–3)	**0.167**	0.116
Daytime dysfunction	1 (0–3)	0 (0–3)	1 (0–3)	**0.229**	0.031*
Global PSQI score	7 (2–18)	6 (2–14)	8 (2–18)	**-0.081**	0.036*
***Multidimensional Scale of Perceived Social Support (MSPSS)***					
Significant Other Subscale	7 (1–7)	7 (1.5–7)	7 (1–7)	-0.034	0.753
Family Subscale	6.75 (1.5–7)	6.75 (1.5–7)	6.5 (2.25–7)	-0.166	0.122
Friends Subscale	4.5 (1–7)	5.5 (1–7)	4.13 (1–7)	-0.188	0.079
MPSS Total Scale	5.58 (1.33–7)	6.25 (1.33–7)	5.54 (1.75–7)	-0.234	0.029*

*MSK* musculoskeletal, *PSQI *Pittsburgh Sleep Quality Index***,**** ESAS* Edmonton Symptom Assessment System

^*^p < 0.05

^a^Mann-Whitney test was used

### Regression analysis

Significant MSD determinants in univariate analysis were evaluated using logistic regression model. Female gender (*p* = 0.013), hypertension (*p* < 0.001), higher scores of pain (*p* = 0.001), fatigue (*p* = 0.012), depression (*p* = 0.014), anxiety (*p* = 0.004), drowsiness (*p* = 0.02), and reduced activity (*p* = 0.029) were significantly associated with MSDs. Similarly, the global PSQI score (0.027), daytime dysfunction (*p* = 0.049), and subjective sleep quality scores (*p* = 0.025) predicted MSDs in the studied HD patients. The use of erythropoietin (*p* < 0.001), calcium (*p* < 0.007) and iron (*p* = 0.01) supplementation and anti-hypertensive drugs (*p* = 0.002) have been all significantly associated with MSDs in the univariate analysis. A multivariable regression model with forward selection procedure was performed including variables with *p*-value < 0.05 in the univariate analysis. At the multivariable regression model, erythropoietin administration (OR [95% CI]) = 5.655 [1.368–23.374], *p* = 0.017) and pain score (OR [95% CI]) = 1.312 [1.013–1.699], *p* = 0.040) were the only independent variables associated with the outcome (Table 3).

**Table 3:** Univariate and multivariate regression analyses to determine predictors of MSK discomfort among the study HD patients (*n* = 94)

	Univariate analysis			Multivariate analysis		
**Variable**	**OR**	**95% CI**	***P***	**OR**	**95% CI**	***P***
*Gender*						
Male	Ref			-	-	-
Female	3.960	1.338–11.720	0.013*	-	-	-
***Clinical characteristics***						
Hypertension	7.700	2.576–23.012	< 0.001*	-	-	-
***Therapeutic data***						
Erythropoitin	8.821	3.143–24.758	< 0.001*	5.655	1.368–23.374	0.017*
Calcium supplementation	3.878	1.438–10.457	< 0.007*	-	-	-
Iron supplementation	4.20	1.419–12.429	0.010*	-	-	-
Antihypertensives	6.565	2.043–21.095	0.002*	-	-	-
***Pain and symptom burden***						
Pain	1.429	1.165–1.753	0.001*	1.312	1.013–1.699	0.040*
Fatigue	1.304	1.059–1.605	0.012*	-	-	-
Depressed	1.302	1.055–1.606	0.014*	-	-	-
Anxiety	1.378	1.110–1.712	0.004*	-	-	-
Drowsiness	1.282	1.040–1.579	0.020*			
**Multidimensional Fatigue Inventory**						
Reduced activity	1.307	1.027–1.663	0.029*	**-**	**-**	**-**
***Pittsburgh Sleep Quality Index***						
Subjective sleep quality	2.023	1.095–3.737	0.025*	-	-	-
Daytime dysfunction	1.741	1.002–3.023	0.049*	-	-	-
Global PSQI score	1.178	1.019–1.362	0.027*	-	-	-

^*^P Value < 0.05%

## Discussion

### Symptom burden

Almost all HD patients have one or more MSK complications, of which muscle cramps, pains and arthralgias are the commonest presentations [29]. Patients with ESKD frequently experience high symptom burden that may impair their health-related quality of life (HRQOL), leading to poor clinical outcomes [1, 2]. An earlier study revealed that patients on HD had, on average, 11 symptoms, denoting high symptom burden, among which fatigue is a common occurrence [30]. Moreover, HD patients prioritized both physical symptoms including insomnia, fatigue, muscle cramps, and mood symptoms including depression and anxiety as the top symptoms necessitating evidence-based interventions for symptom relief [31]. The patient drove symptom prioritization based on the frequency, duration, unpredictability of symptoms as well as their social and financial impacts [31]. The frequency of MSDs has been related to dialysis vintage [6]. The present study identified MSDS in approximately three quarters of the studied sample including patients with ESKD receiving HD who considerably reported pain and fatigue as the most common symptoms. Knee pain (48.9%), low back pain (43.6%), shoulder pain (41.6%), hip/thigh pain (35.1%) and neck pain (35.1%) were the most reported pains. Of note, upper and lower extremity pains have been commonly reported in previous studies [2, 10, 32, 33].

Besides the physical symptoms, the present study revealed a greater tendency for mood symptoms in patients with physical symptoms. The analysis of the modified (ESAS) results revealed that patients with MSDs are more likely to suffer depression and/or anxiety. Mood symptoms are thought to be linked to the prevailing physical symptoms [30, 31]. Chronic pain per se is significantly associated with depression, anxiety, and irritability [34]. Therefore, the amelioration of the most bothersome physical symptoms might help improve depression and anxiety. Depression, anxiety and stress are hypothesized as risk factors for low physical and mental HRQOL [35].

### Demographic and clinical determinants

The present study revealed more MSDs in female rather than male patients conforming to previous research [36–38]. Patients with MSDs were more likely to have hypertension (64.7%) and to be treated with ESAs (*p* < 0.005). The association between hypertension and MSDs in patients with kidney disease is not established and the existing evidence as regard the general population is controversial [39, 40]. Of note, the physical functioning of HD patients was remarkably affected by comorbid conditions [29]. The literature is lacking regarding the effect of ESA on MSDs in patients with CKD. Caravaca et al. found no significant association between the use of ESAs and chronic musculoskeletal pain in patients with CKD [41]. However, this comes contradictory to our results showing high prevalence of MSDs in patients receiving ESAs. Further studies are needed to explain this observation. The present study revealed that patients receiving calcium and iron therapy were more prone to MSDs. Remarkably, the pathophysiological interactions of CKD-MBD including vit D deficiency and consequent hypocalcemia greatly predispose to MSDs in patients with ESKD [1]. Similarly, an association has been reported between decreased vitamin D levels and musculoskeletal pain in the general population [42, 43]. As well, there has been an association between chronic anemia and musculoskeletal disorders [44]. It is noteworthy mention that musculoskeletal symptoms including fatigue, muscle pains, and backache are reported side effects of iron therapy [45].

Although the adequacy of dialysis has been linked to the patients’ quality of life and survival, there is very little research investigating the relationship between adequacy of dialysis and MSDs. The present study showed no significant difference as regards Kt/V or Urea reduction ratio (URR) as measures of dialysis adequacy between patients with and without MSK symptoms. Correspondingly, the indices of dialysis adequacy did not significantly vary between patients with or without chronic pain, anxiety or depression in previous studies [46–48]. Since the adequacy of dialysis relies on measurement of small solute clearance using urea and creatinine, which is only one minor part of the effectiveness of dialysis, it is not necessarily indicating an ‘optimum’ dialysis [49]. Furthermore, the indices of dialysis adequacy may vary in different periods of time, which may not correspond to the patients’ symptoms. Further large-scale clinical trials are thought to be more sensible in solving this dilemma.

### Sleep disturbances and social determinants

Sleep disturbances are frequently encountered among patients receiving dialysis, presenting a significant association with increased morbidity and mortality, however remain under-diagnosed and inadequately managed [50, 51]. In line with this, the present study revealed worse overall PSQI scores in patients with MSDs. Subjective sleep quality, and daytime dysfunction were more significantly encountered. In the landmark study of De Barbieri and Zampieron, polyneuropathy, pain, and morning dialysis shift were predictors of chronic insomnia. Chronic insomnia with daytime dysfunction were more likely reported in hemodialysis than in the general population [52]. Patients on HD feel frustrated and helpless because of poor quality sleep with daytime tiredness from inadequate sleep impairing their health-related quality of life (HRQOL) [50]. Sleep disturbances arising from pain and chronic musculoskeletal impairments predicted frailty in HD patients [53].

Social support is one of the most effective ways to improve the patients’ long-term outcomes and adjustment to illness. It refers to the support given to an individual from other people, and his/her degree of satisfaction with this support. Patients perceiving good social support were found to better cope with their chronic illness, derive more pleasure out of life, and challenge their depressive mood and symptom burden [54, 55]. Family support, marriage and higher incomes represented the main determinants of adequate social support and therefore, a better quality of life [54, 56]. The present study revealed that patients with MSDs significantly perceived less social support from family, friends and overall. Comparably, earlier studies showed that fatigue is commonly encountered in HD patients perceiving less social support [37]. The social impact of chronic hemodialysis represents a major determinant of the patient’s impaired quality of life, however, the association between social support and MSDs is understudied in HD patients. Further studies are still needed to better define this problem.

## Conclusion

MSDs are frequently encountered in HD patients. Female gender, fatigue, depression, anxiety, reduced activity, and poor sleep quality are strongly associated with MSDs in HD patients.

The nephrology community has shown increasing appreciation of the psychosocial wellbeing and health-related quality of life (HRQOL) in hemodialysis patients. Nevertheless, potential therapeutic strategies should be explored to empower patients with MSDs and decrease their suffering. A practical multidisciplinary approach to early identify MSDs is strongly needed. Social programs are warranted to produce sizable and sustained effects on patients’ outcomes. Patient participation with effective behavioral and lifestyle changes is mandatory for reducing the burden of the problem. Randomized controlled trials of interventions and programs targeting the psychosocial determinants of MSK pain should be considered.

### Strengths and limitations of the study

The study provides a comprehensive analysis of MSK problems in HD patients as well as the relationship between those disorders and clinicosocial factors. Nevertheless, there are a number of limitations with this study. It would have been valuable to assess vitamin D levels and investigate their relationship to MSK problems. Furthermore, detailed assessment of mood disorders such as anxiety and depression with validated questionnaires would be more informative. The relatively small number of patients is the most significant limitation of this study. The fact that only HD patients were included makes the absence of a control group a second significant limitation of the study. The cross-sectional design of the study made it difficult to draw conclusions about the links between causes and effects. Lastly, we employed convenience sampling, which could have biased the results. This raises the need for further larger controlled studies exploring the determinants of musculoskeletal disorders in patients with CKD on maintenance HD.

## Data Availability

The datasets used and/or analysed during the current study are available from the corresponding author on reasonable request.

## Abbreviations

CKD-MBD: Chronic kidney disease-Mineral bone disorders
ESKD: End stage kidney disease
HD: Hemodialysis
MFI-20: Multidimensional Fatigue Inventory
HRQOL: Health-related quality of life
MSDs: Musculoskeletal disorders
NMQ-E: Nordic Musculoskeletal Questionnaire
PEW: Protein energy wasting
PSQI: Pittsburgh Sleep Quality Index
